# The determinants of patient care manager role and the implementation of COVID-19 clinical pathway: a cross-sectional study

**DOI:** 10.7717/peerj.13764

**Published:** 2022-07-25

**Authors:** Abdul Aziz Alimul Hidayat, Wen-Ling Chen, Rahimah Mohd Nor, Musrifatul Uliyah, Fatin Lailatul Badriyah, Masunatul Ubudiyah

**Affiliations:** 1 Nursing, University Muhammadiyah of Surabaya, Surabaya, East Java, Indonesia; 2 Nursing, Kaohsiung Medical University Hospital, Kaohsiung, Kaohsiung, Taiwan; 3 Faculty of Health & Life Sciences, Management & Science University, Selangor, Selangor, Malaysia; 4 Nursing, University Muhammadiyah of Surabaya, Surabaya, East Jawa, Indonesia; 5 Nursing, University Muhammadiyah of Surabaya, Surabaya, East Java, Indonesia; 6 Nursing, Universitas Muhammadiyah Lamongan, Lamongan, East Java, Indonesia

**Keywords:** Clinical pathways, Patient care manager, Nurse, COVID-19

## Abstract

**Objective:**

This study aims to determine the factors associated with patient care manager role and the implementation of the clinical pathway among nurses in private hospitals.

**Methods:**

This study was conducted from January–July 2021 using the cross-sectional approach. The sample consisted of 168 nurses working in a private hospital in Surabaya City, East Java, Indonesia. Meanwhile, the data were collected using the Patient Care Manager Role Scale (PCMRS) and analyzed by multiple logistic regression to find the correlation between the variables.

**Results:**

A higher percentage of nurses namely 64.3% had compliance in COVID-19 clinical pathways with an average PCMRS score of 27.81 ± 2.43. Nurses with a high-level patient care manager role level had a significant compliance risk with odds ratio [OR] 440.137, 95% confidence interval [CI] [51.850–3736.184], and *p*-value = 0.000 compared to those with a low role.

**Conclusion:**

The role of patient care manager and compliance with COVID-19 clinical pathways correlated significantly. Based on the results, several actions are needed for the early identification of patient service managers’ roles to ensure compliance with COVID-19 clinical pathways and reduce the number of cases in Indonesia.

## Introduction

The coronavirus pandemic started in Wuhan, China, and spread rapidly to various countries worldwide despite the strategic measures developed (Wu et al., 2020; Wu, Leung & Leung, 2020; Liao, Wang & Kang, 2020). The pandemic has severe challenges and burdens on healthcare systems, particularly among nurses in treating patients ranging from prevention efforts to rehabilitation and considering their health in dealing with the unprecedented risks associated with the outbreak (Buheji & Buhaid, 2020; Billings et al., 2021). By analyzing factors related to the level of patient and nursing safety, health care managers and leaders can provide a comprehensive approach to addressing these issues and providing support to their workforce, while the application of clinical pathways is indispensable (Shanafelt, Ripp & Trockel, 2020; Shahian, 2011).

Globally, the Johns Hopkins Coronavirus Resource Center noted that the death rate in Indonesia reached 53.81 deaths/100k populations (Johns Hopkins Coronavirus Resource Center, 2022). while data from the Ministry of Health in this country stated that there were 114,115 deaths or 2.67%, 4.263.732 positive cases, and 4.114.969 recoveries or 97.1% by the end of 2021 (Santoso et al., 2021; Syuhada et al., 2021). This high mortality rate is presumably caused by various factors, including the comorbidities previously experienced by patients, and also inseparable from service governance namely case management during the pandemic (Zhou et al., 2020; Izcovich et al., 2020; Bhatt et al., 2021). Service continuity is important to improve the quality of service to patients, while the Clinical Pathway is used as a device that acts as quality and cost control, as well as to reduce variations in medical procedures. All tools used are evidence-based with results achieved over a certain period while in hospital; hence, it can be concluded that the clinical pathway is a more effective, efficient, and safe method of patient-centered care (Suh et al., 2020; Xu et al., 2020).

The optimal patient care manager role potentially influences the application of a good clinical pathway for all cases in the hospital. This concept is applied, specifically in the care of COVID-19 patients, and it was predicted that the cure rate will increase (Sfantou et al., 2017; Mianda & Voce, 2018). Managerial activities consist of strategy, culture, and data-centered activities (Parand et al., 2014). The aim of implementing case management through nurse managers is to improve the professionalism and quality of nursing services, which have implications for a good prognosis and potentially reduce mortality (Fabbri, De Maria & Bertolaccini, 2017; Joo & Liu, 2018). However, not all nurses understand their role in the management of COVID-19 disease. This is because they do not have sufficient experience with the clinical pathway in hospital settings (Xu et al., 2020; Gray et al., 2021).The most common problem of clinical care providers is the lack of commitment and responsibility in properly employing and implementing clinical pathways, as well as adjusting to the patients’ needs (Evans-Lacko et al., 2010). This is reinforced by Bao et al. (2016) who stated that implementation of the clinical pathway had a positive impact on the quality of patient care. The important points determine the success of clinical pathway implementation including health professionals, nursing teams, and the hospital context (Jabbour et al., 2018). There is a gap between clinical practice and evidence-based care, which allows other health professions to standardize care. These variations often lead to suboptimal results and potentially increase service costs (Brillstein & Currie, 2020). The implementation of a care manager is important to provide excellent care by paying attention to the quality, safety, and cost of patient care during the treatment or hospitalization period.

Consequently, the role of nurse managers is needed to implement a standardized COVID-19 clinical pathway. The nurse manager needs extensive knowledge and skills to implement their functions and duties, guided by standards maintained by professional organizational case managers (Chandra, Novieastari & Nuraini, 2021). This standard can guide the discharge of responsibilities to clients ethically, effectively, and safely. Recent studies are limited examined the effectiveness and relationship between the implementation of clinical pathways with rapid testing for COVID-19 (Hicks et al., 2021), transition pathway implementation on patients that underwent joint replacement procedures (Xu et al., 2021), as well as bed pathways and length of hospital stay (Leclerc et al., 2021). It is important to note that the application of the COVID-19 Clinical Pathway, particularly in private hospitals in Surabaya, Indonesia, associated with factors and the care manager role has not been investigated. Therefore, this study aims to assess the factors that influence the role of nurse managers and the COVID-19 clinical pathway, as well as to measure the role of nurse managers and their relationship to the COVID-19 clinical pathway.

## Materials and Methods

### Study setting and participants

A simple random sampling method was used to conduct a cross-sectional study in a private hospital with the largest number of COVID-19 patients in Surabaya, Indonesia. The selection of private hospitals was based on being one of the highest Covid referrals in East Java Province, Indonesia. The sample size formula used referred to SK Lwanga & S. Lemeshow based on population size from January 1 to July 31, 2021. A total of 168 qualified and certified nurses to be patient care managers at a private hospital were selected as participants. Furthermore, the inclusion criteria included a nurse manager (patient care manager), who works in a private hospital in the city, and is willing to be a respondent, while the exclusion criteria included those who were not assigned to manage patient care.

### Variables

The independent variables were as follows: age, gender, education level, and length of service. Age was divided into four categories namely 21–25 years, 26–35 years, 36–45 years, and >45 years, while education level was divided into two categories, namely diploma, and Bachelor’s degree or professional nursing education, and gender was classified as either male or female. In addition, the length of service was divided into four including <5, 5–10, and >10 years. Patient Care Manager Role Scale (PCMRS) adopted from the Indonesian National Nurses Association and modified by the researcher was used to determine the role of nursing care managers in various situations. This scale consists of eight items which were rated on a four-point Likert scale namely never, sometimes, often, and always with grades of 1, 2, 3, and 4, respectively. The minimum score on the overall scale is eight, which indicates a low role in the COVID-19 clinical pathway, while the highest score of 32 indicates a good (high) role. Higher and lower levels of the role were determined by scores greater and lower than 24, respectively. A rating scale was also designed to assess the level of COVID-19 clinical pathways application (Suh et al., 2020). The tool had nine items with ratings coded as Yes = 2 and No = 1. The total score was calculated by adding up the individual values for the nine items. The validity and reliability of the Indonesian version instruments have been tested. A certified linguist has translated the questionnaires, and each item assessed by the researcher is related to the conditions, situations, and policies prevailing in Indonesia. Inappropriate items are eliminated without compromising an important component of the questionnaire. Researchers have distributed questionnaires that have been adapted to 30 respondents, and analyzed for questionnaires to assess validity and reliability with Cronbach’s alpha were 0.73 and 0.88, respectively, indicating that it was a valid and reliable instrument.

### Data collection

The participants were registered between January–July 2021 at a private hospital in Surabaya. Data were collected through self-administered questionnaires after the purpose, benefits, disadvantages, and principles of data confidentiality have been clearly explained and understood by all respondents. They were also asked to sign a consent form indicating their willingness to participate in this study, and informed that they can withdraw at any time. Therefore, they completed the questionnaire only after giving their consent.

### Data analyze

To manage and analyze the data, the IBM Statistical Package for Social Science (SPSS) version 23 (SPSS Inc., Chicago, IL, USA) was used. Descriptive analyses were used to analyze the frequency for the categorical variables, while the mean and standard deviation were utilized for the continuous variables. Furthermore, multiple logistic regression was performed to determine the significance of the variables. The significance value was *p* < 0.05, and the adjusted odds ratio (AOR) had a 95% confidence interval (CI).

### Ethical consideration

This study was approved by the scientific study and ethics committee at one of the Universities in Surabaya, Indonesia, with a number IRB. 005071021. The first page of the questionnaire stated the aim, benefits, objectives as well as the agreement of participation. The participants gave their consent before filling in the questionnaire, also, participation was voluntary, and this study did not pose any potential harm physically or mentally.

## Results

### Characteristics of participants and their association with clinical pathways and patient care manager role

The demographic data showed that more than half of the participants or 59.5% were 25–35 years old, 108 or 64.3% were females, 71.4% had a diploma in nursing education, and 42.9% had worked for more than 10 years. Regarding the level of clinical pathway applications, female nurses (66.7%) and age groups of 25–35 years (40.7%) were in the high category with 66.7% and 40.7% respectively. It also inline a high level of nurse’s care manager role with numbers 66.0% and 42.2%, respectively. Furthermore, diploma education level and length of service >10 years showed good application with 66.7% and 59.3%. Length of service >10 years also have the high level of patient care manager role (62.0%). Based on the univariate analysis results, nurses with an age of 25–35 years old were 4.030 times more likely to have a high level of clinical pathways implementation than those with an age <25 years old (OR: 4.030; 95% CI [0.591–27.469]). Nurses with length of service >10 years were 2.665 times more likely to have a high level of clinical pathways implementation than those with less than 5 years (OR: 2.665; 95% CI [0.829–8.567]). Regarding the level of patient care manager role, nurses with >10 years of works were 6.941 times more likely to high level of nurse’s care manager role (OR: 6.941; 95% CI [3.820–12.613]). Another finding shows that nurses with an age of 25–35 years old were 4.188 times more likely to have high level of patient care manager role (OR: 4.188; 95% CI [3.781–17.728]) (Table 1).

**Table 1:** Participant characteristics (*n* = 168).

Variable	Frequency		Level of clinical pathways implementation				OR (95% CI)	*p*-Value	Level of patient care manager role				OR (95% CI)	*p*-Value
			Low (*n* = 60)		High (*n* = 108)				Low (*n* = 71)		High (*n* = 97)			
	*n*	%	*n*	%	*n*	%			*n*	%	*n*	%		
Age							4.030 [0.591–27.469]	0.155					4.188 [3.781–17.728]	0.126
<25 years	4	2.4	0	0.0	4	3.7			4	5.6	0	0.0		
25–35 years	100	59.5	56	93.3	44	40.7			59	83.0	41	42.2		
36–45 years	44	26.2	4	6.67	40	37.0			8	11.4	36	37.1		
>45 years	20	11.9	0	0.0	20	18.5			0	0.0	20	20.7		
Gender							0.253 [0.055–1.159]	0.077					1.895 [0.836–4.296]	0.062
Female	108	64.3	36	60.0	72	66.7			44	62.0	64	66.0		
Male	60	35.7	24	40.0	36	33.3			27	38.0	33	34.0		
Educational level							0.383 [0.089–1.648]	0.198					2.256 [0.959–5.307]	0.000
Diploma	120	71.4	48	80.0	72	66.7			52	73.2	68	70.1		
Bachelors/Nurse Profession	48	28.6	12	20.0	36	33.3			19	26.8	29	29.9		
Length of Service							2.665 [0.829–8.567]	0.100					6.941 [3.820–12.613]	0.000
<5 years	40	23.8	28	46.7	12	11.1			35	49.2	5	5.10		
5–10 Years	56	33.3	24	40.0	32	29.6			24	33.8	32	32.9		
>10 years	72	42.9	8	13.3	64	59.3			12	17	60	62.0		

**Note:**

CI, Confidence Interval; OR, Odd Ratio.

### The role of patient care manager

The results showed that most nurses or 64.3% had a high score for the role of patient care manager, with an average of 24.00 ± 5.65. Based on the analysis, three factors dominate the high role of nurses in the clinical pathway application namely facilitating communication and coordination between members of the health care team (95.8%), educating COVID-19 patients (94.6%) and empowering the patients to solve problems (92.9%). Meanwhile, assisting clients in transitioning safe care to the next most appropriate level with 59.5% was in the low category (Table 2).

**Table 2:** The role of patient care manager in the implementation of COVID-19 clinical pathways (*n* = 168).

Patient care manager role	Score Mean ± SD	Low		High	
		*n*	(%)	*n*	(%)
Comprehensive assessment of health and psychosocial needs of COVID-19 patients	2.92 ± 0.77	56	33.3	112	66.7
Planning with COVID-19 patients, their families and caregivers, doctors in charge, other service providers,	2.30 ± 0.91	64	38.1	104	61.9
Facilitating communication and coordination between members of the health care team, and involving COVID-19 patients in the decision-making process	2.91 ± 0.27	7	4.2	161	95.8
Educating COVID-19 patients, their families or caregivers and members of the health care team	3.48 ± 0.75	9	5.4	159	94.6
Empowering COVID-19 clients to solve problems	3.46 ± 0.75	12	7.1	156	92.9
Promoting the proper use of health care services and striving to improve the quality of care	2.71 ± 1.33	64	38.1	104	61.9
Assisting clients in transitioning safe care to the next most appropriate level	2.71 ± 1.30	68	40.5	100	59.5
Advocacy between client and cost guarantor to facilitate positive outcomes for clients, health team and cost guarantor.	2.83 ± 0.75	67	39.9	101	60.1
Total score	24.00 ± 5.65				

### The role of patient care managers and compliance with COVID-19 clinical pathways

The results showed that 64.3% and 35.7% of patient care manager was in the high and low category, respectively. This implies that the risk of implementing clinical pathways for COVID-19 was significantly good/high as demonstrated by *p* < 0.05.

Based on the results, the correlation between the levels of COVID-19 clinical pathway implementation with patient care manager role was significant (*p* < 0.05). The scores for the high and low-level role were 27.81 ± 2.43 and 17.33 ± 2.29, respectively (Table 3). According to logistic regression analysis, the results consistently showed that nurses with a high level of patient care manager role had a good possibility to implement clinical pathways (AOR: 440.137; 95% CI [51.850–3,736.184]) (Table 4).

**Table 3:** The average score of patient care manager role and their relationship with the implementation of the COVID-19 clinical pathway (*n* = 168).

Variables	Health protocol implementation	
	Mean + SD	*p*-value
Patient care manager role		
Low (*n* = 60)	17.33 ± 2.29	0.000*
High (*n* = 108)	27.81 ± 2.43	

**Note:**

* Significant value at *p*, 0.05.

**Table 4:** Factors associated with the implementation of clinical pathways for COVID-19 from stepwise logistic regression analysis (*n* = 168).

Variable	*n* (%)	AOR (95% CI)	OR (95% CI)	*p*-value
Levels of patient care manager role				
Low	60 (35.7)	440.137 [51.850–3,736.184]	8.812 [0.000–0.000]	0.000*
High	108 (64.3)			

**Note:**

* Significant value at *p*, 0.05.

## Discussion

It is important to consider the role of nursing care managers working in private hospitals, as this is closely related to improving patient safety, particularly compliance with the implementation of the COVID-19 clinical pathway. Previous studies were conducted in relation to this subject (Gutenstein, Pickering & Than, 2019) but none were linked to the role of nurses. The demography data showed that the high factors affecting the level of clinical pathways implementation were the length of service above ten years, and also on the young adult group, who tend to be more active and have ease in remembering and practicing clinical pathways. In addition, the application of clinical pathways is also higher in the female group and in diploma-level nurses. Therefore, it was concluded that a long service period can affect the level of experience and knowledge of nurses in handling clinical cases in hospitals.

From this study we can find the newest findings, there are three main factors that dominate the role of the nursing manager, that are (1) the nurse’s ability to facilitate communication and coordination between members of the health care team, and involving COVID-19 patients in the decision-making process; (2) their ability to educate COVID-19 patients, their families or caregivers and members of the health care team and (3) their ability to empower COVID-19 patients to solve problems.

The results indicated that almost half of the nurses’ roles had facilitated communication and coordination between members of the health care team involving COVID-19 patients in the decision-making process. This is due to the several activities such as performing primary functions of assessment, planning, facilitating, and advocacy which were achieved through collaboration with other health professionals involved in client care. In addition, the case manager must be able to liaise between patients and other health teams to meet their needs, defend their interests and help patients understand all information and health efforts provided by the health team (Quigley-Stickney, 2021; Baker, Nelson & Krsnak, 2021; Schmid & Downey, 2022). To optimize the implementation of the COVID-19 clinical pathway, assistance is needed by the head of the room to improve the compliance with nursing care. Mentoring is a follow-up activity from the supervision process carried out by the head of the room as the manager of patient care. It is an important instrument for hospitals and nurses because, with programmed assistance, they can gain knowledge, understanding, direction, and motivation (Coventry & Hays, 2021; Kurland & Campagna, 2022). With an optimal mentoring program, nurses can be more professional in carrying out their duties and obligations, but the ability of the head of the room in providing mentoring must also be improved (Elcock & Sookhoo, 2007; Chopra et al., 2021; Kurland & Campagna, 2022). This is consistent with a previous study which stated that a patient service manager has an important role in providing motivation, effective communication, and guidance to nurses (Cathcart, 2020).

Furthermore, nurses have important roles in educating COVID-19 patients, their families or caregivers, and members of the health care team. These results are similar to a previous study which showed that education or providing information to teams and families can improve the prevention of COVID-19 cases and disease control (Eddy, Jordan & Stephenson, 2016). By providing a chance for families and teams to be involved in the case management process, hospitals can help improve the capacity and quality of service provided in such conditions.

These results find show that another important role that must be conducted by nurses as care managers are to empower patients to solve their problems. This finding is corelate with the recent study by Mata-Cervantes, Clay & Baxter (2016) that a good level of patient empowerment will provide positive and broad changes in health care. Some of the impacts felt by health workers and patients when empowerment is applied to their patients are more obedient and care about their health status, so this can improve the quality of care (Mata-Cervantes, Clay & Baxter, 2016). By empowering patients to take shared responsibility for the management of their COVID-19 condition and solve their problems, they may not only promote good medical outcomes but improve their psychological well-being because they are involved in every nursing process.

The results indicate that the high level of nursing care managers’ role has a significant relationship with the implementation of the COVID-19 clinical pathway. This is because patient service managers play a role in providing motivation and guidance to nurses by using effective and easy-to-understand communication. Effective communication helps a manager in conveying information for a better understanding and utilization by nurses (Cathcart, 2020; Marwa, 2021). This study provides new information indicating that due to the proportion of study respondents.

Based on the proportion of the respondents with the low role of patient care manager level, efforts that address the salient factors are needed to increase the implementation of COVID-19 clinical pathways among nurses in private hospitals. Given that this study was limited to areas in East Java, future studies covering more areas are needed to further explicate and strengthen the factors that affect nurses’ role in increasing the implementation of COVID-19 clinical pathways in a private hospital.

## Conclusions

More than half of the participants had a high patient care manager role, with only a few at low levels. Factors that affect patients’ care manager role are facilitated communication and coordination between members of the health care team, education and empowering patients to solve their problems. Length of work and education level also affect the level of clinical pathway and nurse care manager role. Nurses with high-level roles had a good implementation of COVID-19 clinical pathways and this result requires serious attention in private hospitals. This implies the in-house training program on COVID-19 clinical pathways for nurses as patient service managers are important and must be sustained. Furthermore, monitoring and information boards need to focus on increasing the role of patient service managers in implementing clinical pathways to overcome failures in handling COVID-19.

## Supplemental Information

10.7717/peerj.13764/supp-1 Supplemental Information 1 Raw data.

10.7717/peerj.13764/supp-2 Supplemental Information 2 Questionnaire in English.

10.7717/peerj.13764/supp-3 Supplemental Information 3 Questionnaire in Indonesia.

## Additional Information and Declarations

### Data Availability

The following information was supplied regarding data availability: The raw measurements are available as a Supplemental File.
