# Supplementary material for: The determinants of patient care manager role and the implementation of COVID-19 clinical pathway: a cross-sectional study
Source: PeerJ. 2022 Jul 25;10:e13764. doi: 10.7717/peerj.13764 (PMC9332306; doi:10.7717/peerj.13764)
Supplement: Supplemental Information 2 [file peerj-10-13764-s002.docx]

**QUESTIONNAIRE**

Respondent Number :

Instruction :

Fill in the questions below, if there is a choice, circle one

1. Respondent characteristics

Age : ……….Year

Gender : 1. Female

2. Male

Educational Level : 1. Diploma

2. Bachelors/Nurse Profession

Lengt of Work :..........Year

1. Implementation of Clinical Pathway on COVID-19 in hospitals

(Observation)

| No | Question | Yes | No |
| --- | --- | --- | --- |
| 1 | Do nurses record the medical records of Covid-19 patients every day? |  |  |
| 2 | Do nurses do assessments on COVID-19 patients every day? |  |  |
| 3 | Do nurses carry out developmental diagnoses on Covid-19 patients every day |  |  |
| 4 | Do nurses observe vital signs on Covid-19 patients every day? |  |  |
| 5 | Do nurses monitor oxygen saturation (a sign of hypoxia) in Covid-19 patients every day? |  |  |
| 6 | Does the nurse maintain the airway in Covid-19 patients at all times |  |  |
| 7 | Do nurses keep hands and feet clean, especially after coughing in Covid-19 patients? |  |  |
| 8 | Do nurses maintain a normal temperature, use appropriate therapy to reduce excess metabolic needs in COVID-19 patients |  |  |
| 9 | Do nurses provide health education to patients and families of COVID-19 patients regarding disease transmission, diagnostic examinations and the disease process? |  |  |

2. The role of the Nursing Manager in the implementation of clinical pathways for COVID-19 patients

| No | Question | never | Sometimes | often | always |
| --- | --- | --- | --- | --- | --- |
| 1 | Does the nurse manager carry out a comprehensive assessment of the patient's health and psychosocial needs of COVID-19 patients? |  |  |  |  |
| 2 | Do nurse managers plan together with Covid-19 patients, their families and caregivers, doctors in charge, other service providers |  |  |  |  |
| 3 | Do nurse managers facilitate communication and coordination between health team members who involve COVID-19 patients in the decision-making process |  |  |  |  |
| 4 | Do nurse managers Educate COVID-19 patients, their families or caregivers and members of the healthcare team |  |  |  |  |
| 5 | Are nurse managers Empowering covid-19 clients to solve problems |  |  |  |  |
| 6 | Do nurse managers encourage appropriate use of health services and strive to improve the quality of care? |  |  |  |  |
| 7 | Does the nurse manager assist the client in transitioning safe care to the next most appropriate level? |  |  |  |  |
| 8 | Does the nurse manager assist the client in transitioning safe care to the next most appropriate level? |  |  |  |  |
