# Supplementary material for: The determinants of patient care manager role and the implementation of COVID-19 clinical pathway: a cross-sectional study
Source: PeerJ. 2022 Jul 25;10:e13764. doi: 10.7717/peerj.13764 (PMC9332306; doi:10.7717/peerj.13764)
Supplement: Supplemental Information 3 [file peerj-10-13764-s003.docx]

**LEMBAR KUESIONER**

No Responden :

Petunjuk :

Isilah Pertanyaan dibawah ini, jika ada pilihan lingkari salah satu.

1. Karakteristik Responden

Usia : ……….Tahun

Jenis Kelamin : 1. Perempuan

2. Laki-Laki

Tingkat Pendidikan : 1. Diploma

2. Sarjana/Profesi Perawat

Lama Bekerja :..........tahun

1. Implementasi Clinical Patway pada COVID-19 di rumah sakit (Observasi)

| No | Pertanyaan | Ya | Tidak |
| --- | --- | --- | --- |
| 1 | Apakah perawat mencatat rekam medis pasien Covid-19 setiap hari |  |  |
| 2 | Apakah perawat melakukan assessment pada pasien covis-19 setiap hari |  |  |
| 3 | Apakah perawat melakukan diagnosis perkembangan pada pasien Covid-19 setiap hari |  |  |
| 4 | Apakah perawat melakukan observasi tanda vital pada pasien covid-19 setiap hari |  |  |
| 5 | Apakah perawat melakukan monitoring saturasi oksogen (tanda dari hipoksia) pada pasien covid-19 setiap hari |  |  |
| 6 | Apakah perawat mempertahankan jalan nafas pada pasien covid-19 setiap saat |  |  |
| 7 | Apakah perawat menjaga kebersihan tangan dan kaki khususnya setelah batuk pada pasien covid-19 |  |  |
| 8 | Apakah perawat mempertahankan suhu normal, menggunakan terapi yang tepat untuk mengurangi kebutuhan metabolisme berlebih pada pasien covid-19 |  |  |
| 9 | Apakah perawat Memberikan pendidikan kesehatan kepada pasien dan keluarga pasien COVID-19 mengenai penularan penyakit, pemeriksaan diagnostik dan proses penyaki |  |  |

2. Peran Manajer Keperawataan dalam implementasi clinical patways pada pasien COVID-19

| No | Pertanyaan | Tidak Pernah | Kadang-kadang | Sering | Selalu |
| --- | --- | --- | --- | --- | --- |
| 1 | Apakah manajer perawat melakukan Asesmen komprehensif terhadap kesehatan pasien dan kebutuhan psikososial pasien covid-19 |  |  |  |  |
| 2 | Apakah manajer perawat melakukan rencana bersama engan pasien Covid-19, keluarga dan pengasuhnya, dokter penanggung jawab, penyedia layanan lainnya |  |  |  |  |
| 3 | Apakah manajer perawat memfasilitasi komunikasi dan koordinasi antar anggota tim kesehatan yang melibatkan pasien COVID-19 dalam proses pengambilan keputusan |  |  |  |  |
| 4 | Apakah manajer perawat Mendidik pasien COVID-19, keluarga atau pengasuh mereka dan anggota tim perawatan kesehatan |  |  |  |  |
| 5 | Apakah manajer perawat Memberdayakan klien covid-19 untuk menyelesaikan masalah |  |  |  |  |
| 6 | Apakah manajer perawat Mendorong penggunaan yang tepat dari layanan kesehatan dan berusaha untuk meningkatkan kualitas perawatan |  |  |  |  |
| 7 | Apakah manajer perawat Membantu klien dalam mentransisikan perawatan yang aman ke tingkat berikutnya yang paling tepat |  |  |  |  |
| 8 | Apakah manajer perawat Membantu klien dalam mentransisikan perawatan yang aman ke tingkat berikutnya yang paling tepat |  |  |  |  |
